# Ethnographic Methods, Music, and Aging

**DOI:** 10.1093/geroni/igaa057.1864

**Published:** 2020-12-16

**Authors:** Amanda Couve, Joseph Kotarba

**Affiliations:** 1 Encompass Health - Hospice, San Antonio, Texas, United States; 2 Texas State University, San Marcos, Texas, United States

## Abstract

Qualitative methods are proving to be important tools for studying the multi-faceted experience of living with aging, with a focus on the arts. Ethnographic methods are productive ways to discover and examine music in everyday life. Systematically studying the normal and often taken-for-granted ways aging adults experience music, in the full range of settings where they can be found, can abide by the first rule of translational science research: to design and conduct research in order to facilitate the efficient and timely development and application of clinical and caring interventions. This presentation will review a series of ethnographic studies of music experiences in residential facilities, dementia respite groups, family, and hospice. We will suggest ways to apply findings from these studies to enlighten volunteer hospice workers’ protocols for care.

